# Dietary Fiber Modulates the Fermentation Patterns of Cyanidin-3-*O*-Glucoside in a Fiber-Type Dependent Manner

**DOI:** 10.3390/foods10061386

**Published:** 2021-06-16

**Authors:** Zixin Yang, Ting Huang, Ping Li, Jian Ai, Jiaxin Liu, Weibin Bai, Lingmin Tian

**Affiliations:** 1 Department of Food Science and Engineering, Jinan University, Guangzhou 510632, China; yangzixin121@163.com (Z.Y.); huangting081@163.com (T.H.); aijian23@163.com (J.A.); liujiaxin9898@163.com (J.L.); 2 Guangdong Engineering Technology Center of Food Safety Molecular Rapid Detection, Institute of Food Safety and Nutrition, Jinan University, Guangzhou 510632, China; liping5477@163.com (P.L.); baiweibin@163.com (W.B.); 3 Department of Food Science and Engineering, College of Food Science, Shanghai Ocean University, Shanghai 201306, China

**Keywords:** dietary fiber, fermentation patterns, cyanidin-3-*O*-glucoside, gut microbiota

## Abstract

The interactions between cell-wall polysaccharides and polyphenols in the gastrointestinal tract have attracted extensive attention. We hypothesized that dietary fiber modulates the fermentation patterns of cyanidin-3-*O*-glucoside (C3G) in a fiber-type-dependent manner. In the present study, the effects of four dietary fibers (fructose-oligosaccharides, pectin, β-glucan and arabinoxylan) on the modulation of C3G fermentation patterns were investigated through in vitro fermentation inoculated with human feces. The changes in gas volume, pH, total carbohydrate content, metabolites of C3G, antioxidant activity, and microbial community distribution during in vitro fermentation were analyzed. After 24 h of fermentation, the gas volume and total carbohydrate contents of the four dietary-fiber-supplemented groups respectively increased and decreased to varying degrees. The results showed that the C3G metabolites after in vitro fermentation mainly included cyanidin, protocatechuic acid, 2,4,6-trihydroxybenzoic acid, and 2,4,6-trihydroxybenzaldehyde. Supplementation of dietary fibers changed the proportions of C3G metabolites depending on the structures. Dietary fibers increased the production of short-chain fatty acids and the relative abundance of gut microbiota *Bifidobacterium* and *Lactobacillus*, thus potentially maintaining colonic health to a certain extent. In conclusion, the used dietary fibers modulate the fermentation patterns of C3G in a fiber-type-dependent manner.

## 1. Introduction

Anthocyanins, a class of phenolic compounds belonging to the flavonoid group, are mainly found in the epidermal tissues of plants, such as vegetables, fruits, and flowers [1]. Anthocyanins are responsible for organoleptic colors such as red, purple, and blue [2]. Cyanidin-3-*O*-glucoside (C3G) is one of the most widely distributed anthocyanins in fruits [3], accounting for 94% of the total anthocyanin content in blackberries [4,5]. C3G serves as key antioxidant. Anthocyanins are glycosides of polyhydroxy and polymethoxy derivatives of 2-phenylbenzopyrylium or flavylium salts [2]. These compounds have a high antioxidant capacity [6] and the ability to prevent a variety of chronic diseases associated with inflammation [7,8] and improve the colon environment [9].

Dietary phenols, such as C3G, are substrates for a variety of enzymes in the liver, small intestine, and colon [10,11], mainly enzymes produced by gut microbiota. The colon is therefore an active site for C3G metabolism. When C3G is present in the gastrointestinal tract (GIT), colonic microbiota decompose them into a variety of bioactive substances and phenolic acids, such as protocatechuic acid (PCA) and 2,4,6-trihydroxybenzaldehyde (TB) [12], derived from the parent compound [12,13,14]. C3G is likely to primarily exert its influence through C3G metabolites [15]. In addition, in our previous study [16], we found that C3G plays a role in regulating the prebiotic activity of the microbial composition in rats. Another study found that the fermentation of C3G stimulated the proliferation of *Bifidobacteria* and *Lactobacillus*; both effects can affect intestinal health [17].

In addition to polyphenols, dietary fiber could modulate gut microbiota in a structure-dependent manner, releasing short-chain fatty acids (SCFAs) in the GIT. In recent years, the interactions between anthocyanins and dietary fibers have attracted extensive attention. A significant increase in plasma antioxidant capacity was found 8 h after the acute ingestion of high dietary fiber and polyphenols (antioxidant capacity determined by ABTS^•+^ and FRAP assays) [18]. The previous study has suggested that certain pectin fractions can increase C3G stability during in vitro digestion [19] in upper GIT, and consequently change the substrates for microbiota in the colon. The interactions between anthocyanins and dietary fibers could benefit gut health. However, how this interaction affects the fermentation of dietary fiber and C3G is still unclear, especially in terms of how different dietary fibers modulate C3G fermentation patterns depending on fiber type.

In this study, we investigated the interplay of C3G with four different dietary fibers during in vitro fermentation. We used in vitro fermentation model to determine the effects of different dietary fibers (β-glucan, arabinoxylan, fructo-oligosaccharide, and pectin) on the C3G metabolites, antioxidant capacity, SCFAs, and the microbiota composition in the colonic environment.

## 2. Materials and Methods

### 2.1. Materials

Oat β-glucan (BG) and wheat arabinoxylan (AX) were purchased from Shaanxi Pioneer Biotech Co., Ltd. (Xi’an, Shaanxi, China). Pectin (PEC) with 71% degree of esterification was provided by Herbstreith & Fox (Neuenbürg, Germany). Fructo-oligosaccharide (FOS) was obtained from Shanghai Yuanye Bio-Technology Co., Ltd. (Shanghai, China). Cyanidin-3-*O*-glucoside (C3G) was extracted and purified from black soybean peels (Anhui province, China) according to the method we described before [20,21]. The purity of the C3G was 95%.

### 2.2. In Vitro Fermentation Model

#### 2.2.1. Medium

The culture medium was based on the modified standard ileal efflux medium (SIEM), as previously described [22]. Per liter, the medium contained the following: 4.5 g NaCl, 2.5 g K_2_HPO_4_, 0.45 g CaCl_2_·2H_2_O, 0.5 g MgSO_4_·7H_2_O, 0.005 g FeSO_4_·7H_2_O, 0.05 g ox bile, 0.01 g haemin, 0.4 g cystein, 3 g bactopeptone, and 3 g casein. We then added 1 mL of a vitamin mixture, per liter of the SIEM contained: 1 mg menadione, 2 mg D-biotin, 0.5 mg vitamin B-12, 10 mg pantothenate, 5 mg nicotinamide, 5 mg *para*-aminobenzoic acid, and 4 mg thiamine.

#### 2.2.2. Fecal Inoculum

Fecal samples were obtained from four healthy donors aged 22 to 23 years (mean age 23) who did not have any intestinal diseases and had not been treated with antibiotics for the previous three months. The donors’ mean Body Mass Index (BMI) was 26.3 kg/m^2^ (range: 22.8–35.1). Fecal samples were collected in a centrifuge tube with a sterile phosphatic buffer saline (PBS); then they were swirled and centrifuged (5000× *g*, 10 min). The mixture was diluted with a sterile PBS buffer to 0.1 g/mL to obtain the final fecal inoculum.

#### 2.2.3. In Vitro Fermentation

The FOS, PEC, BG, and AX were each mixed with the C3G (CF, CP, CB, and CA groups), and each of the four mixtures was added to the SIEM culture medium as the substrates. The final fermentation system contained a 10 mg/mL concentration of dietary fiber and a 1 mg/mL concentration of C3G. Another experimental group supplemented only with a 1 mg/mL concentration of C3G (C group) was designed. The medium without any dietary fiber or C3G was used as blank control (N group). The mixture (3.6 mL) of medium and fermentation substrate was placed into an anaerobic fermentation tube; then, under anaerobic conditions, we added 0.4 mL of fecal inoculum. The in vitro fermentation was carried out in a vibrating incubator (37 °C, 100 rpm), and the fermentation fluids were collected and stored in a refrigerator, at −80 °C, after 0, 0.5, 1, 2, 4, 12, and 24 h. Each group was repeated three times in this study.

### 2.3. Antioxidant Capacity of the Fermentation Samples

The DPPH free-radical scavenging ability of the fermentation samples was determined according to a previous method [23].

The determination of ABTS radical cation scavenging activity was based on the method described before [24].

### 2.4. Gas Volume and pH

The measurement of gas volume during fermentation was based on a previous study [25]. This was done by emptying the gas in a syringe, and then inserting the syringe needle into the rubber stopper of the anaerobic bottle, allowing the gas produced in the anaerobic bottle to drain into the syringe. After fermentation, the volume of produced gas in each syringe was recorded.

The pH value was performed at 0, 4, 12, and 24 h after starting the colonic incubation, using a pH meter (PHS-3C, Leici, China) with a pH electrode (PY-P22-2S, Sartorius, Germany).

### 2.5. Determination of Total Carbohydrate Contents

Total carbohydrate content was determined according to the phenol–sulfuric acid method. We mixed 0.45 mL of 72% concentrated sulfuric acid and lyophilized 1 mL fermented sample in a test tube; then we heated the mixture at 30 °C for 1 h. We then added 4.95 mL of distilled water and heated the mixture at 100 °C for 3 h to obtain the hydrolyzed liquid for testing. The hydrolyzed sample was diluted at an appropriate dilution factor. A total of 200 µL of the diluted solution and 800 µL of a 2.5% phenol solution were mixed into the test tube. Then, 2.5 mL of concentrated sulfuric acid was added to the tube, and the mixture was mixed well and cooled to room temperature. The absorption value was read at 490 nm, and the total carbohydrate concentration of the fermentation liquid was calculated with a linear regression equation established for glucose.

### 2.6. Analysis of C3G and Metabolites

The High-Performance Liquid Chromatography (HPLC) system (Thermo U3000, USA) was used for the chromatographic separation of the C3G and its metabolites in all sample groups during fermentation. Reversed-phase chromatography was performed with a C18 column (4.6 × 250 mm, 5 μm; Thermo, Waltham, MA, USA). The mobile phase was a 2% formic acid aqueous solution (A) and acetonitrile (B). The gradient elution program was as follows: 0–34 min, 6–30% B; 34–35 min, 30–90% B; 35–40 min, 95–95% B; 40–41 min, 95–95% B; and 41–45 min, 6–6% B.

The Ultraviolet (UV) absorption intensity of the eluent was read at 280 and 520 nm. Compared with the peak area of standards for C3G and C3G metabolites, the contents of the C3G and C3G metabolite at different fermentation times were calculated.

### 2.7. DNA Extraction and 16S rRNA Sequence

Total genomic DNA was extracted by using a MagPure Soil DNA KF Kit (Catalogue No. D6356-02), and the manufacturer’s instructions were followed. Quality and quantity of DNA were verified with NanoDrop and agarose gel. Then, the sample was diluted to 1 ng/µL and stored at −20 °C until further processing. Two primer pairs, 343F-5′-TACGGRAGGCAGCAG-3′ and 798R-5′-AGGGTATCTAATCCT-3′, were conducted to amplify the V3/V4 region (343–798) of the 16S rRNA gene. For library construction, a 30-µL PCR mixture was prepared as follows: 10–50 ng of DNA template, 1 µL of the forward and reverse PCR primers (5 pmol/μL each), 15 µL 2× Gflex PCR buffer (Takara), and 0.6 μL of Tks Gflex DNA polymerase (1.25 U/μL, Takara). An appropriate volume of double-distilled water (ddH_2_O) was added to bring the volume up to 30 μL. The PCR program was as follows: initial denaturation at 94 °C for 5 min, followed by 26 cycles of denaturation at 94 °C for 30 s, annealing at 56 °C for 30 s, extension at 72 °C for 20 s, and a final extension at 72 °C for 5 min.

### 2.8. Analyses of SCFAs

SCFA contents, including acetic, propionic, butyric, valeric, isobutyric, and isovaleric acids in the fermentation fluids, were analyzed by using gas chromatography (GC; Shimadzu G2010Plus, Kyoto, Japan) on a DB-FFAP chromatographic capillary column (30 m × 0.530 mm × 1.00 μm; Agilent, Santa Clara, CA, USA), with calculated standard curves based on the method described in Tian et al. [26], with minor modifications. A total of 200 μL of the fermentation samples was mixed with 200 μL of 0.3 mg/mL 2-ethylbutyric acid and 50 μL of 0.15 M oxalic acid. The mixture was filtered at 0.22 μm into a vial for analysis.

### 2.9. Statistical Analysis

GraphPad Prism 8.0 (San Diego, CA, USA) was used for the graphic presentation. Data other than microbial diversity and composition were expressed as means ± standard deviation (SD), and the differences among all the groups were tested with a one-way analysis of variance (ANOVA) with multiple post hoc comparisons. A value of *p* < 0.05 was considered to be statistically significant. Chao1, Shannon, and Simpson indices were applied to evaluate alpha diversity. Weighted-UniFrac distance was used to evaluate beta diversity in different fermented samples.

## 3. Results and Discussion

### 3.1. Antioxidant Capacity

**Figure 1:**
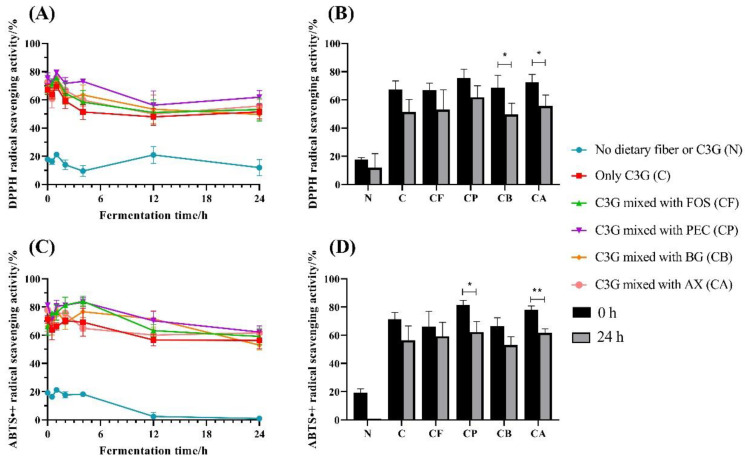
Changes in antioxidant activity, DPPH (**A**), and ABTS^•+^ (**C**) radical scavenging rate for C3G mixed with different polysaccharides during in vitro fermentation; differences in DPPH (**B**) and ABTS^•+^ (**D**) radical scavenging rate in vitro fermentation for 0 and 24 h. Asterisks indicate significant difference between fermentation for 0 and 24 h, according to an ordinary one-way ANOVA: * (*p* < 0.05) and ** (*p* < 0.01). Values are means ± SD for *n* = 3.

The results for DPPH and ABTS^•+^ scavenging ability over the 24 h fermentation period are shown in Figure 1A,C. The antioxidant activity of each group decreased after 24 h in vitro fermentation; however, the decrease was not significant in every group. This may be due to the lack of metabolic absorption pathways in our simulated colon process, causing the phenolic substances to remain in the fermentation broth after degradation. Compared with 0 h, the DPPH radical scavenging power decreased 23.4% for C, 20.6% for CF, 17.9% for CP, 27.8% for CB (*p* < 0.05), and 23.2% for CA (*p* < 0.05), respectively (Figure 1B). The ABTS^•+^ scavenging power of the fermentation samples showed a similar trend (Figure 1C,D). The antioxidant activity in the samples indicated that the mixtures of C3G with FOS, PEC, or AX could all retain the antioxidant active components in the fermentation broth to varying degrees. C3G and C3G metabolites are considered to be major contributors to antioxidant capacity, due to their chemical structure [2]. The uronic acids–rich fractions in polysaccharides have higher antioxidant activities and are significant for the biological activities of polysaccharides [27]. Pectin contains the greatest amounts of uronic acids among the four dietary fibers, so the CP group had the strongest antioxidant activity in vitro. The mixture of C3G and BG had an antagonistic effect on antioxidant activity, and this might be due to its structure and provided dose, as well as the strong hydrogen bonds between the two metabolites [28].

### 3.2. Total Gas Production and pH

**Figure 2:**
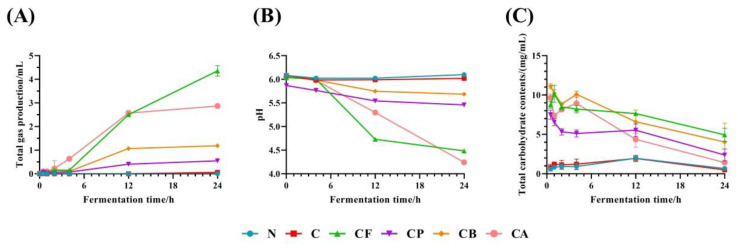
Cumulative gas production (**A**), pH changes (**B**), and total carbohydrate contents (**C**) during in vitro fermentation for C3G and different polysaccharides.

The total gas production for C3G and the different polysaccharides during in vitro fermentation is shown in Figure 2A. The cumulative gas production of the groups with added polysaccharides was higher than that of group N and group C. Cumulative gas production increased gradually with increasing fermentation time. The CP, CB, and CA groups had no obvious gas-production changes after 12 h, while the CF group still showed an increasing trend in gas production. By the end of the 24 h fermentation time, the volume of cumulative gas production for the CF, CP, CB, and CA groups had reached 4.36 ± 0.19, 0.55 ± 0.07, 1.19 ± 0.05, and 2.87 ± 0.05 mL, respectively. Fermentation of FOS from using pig fecal microbiota produced a higher volume than that of the other carbohydrates used as substrates [29]. After 24 h in vitro fermentation, the results for gas production with different polysaccharide substrates were similar to those of previous studies [30].

The fermentation of carbohydrates by human gut microbiota usually produces gases. Production of H_2_ is often necessary for the cycling of NAD^+^/NADH during fermentation, and CO_2_ is released whenever decarboxylation occurs [31]. C3G did not have sufficient carbohydrates for the fermentation by gut microbiota; therefore, the gas volume of C group was very low (Figure 2A). The chemical composition of the prebiotics and the composition of the microbiota are related to the amount of produced gas during fermentation [31]. A previous study showed that inulin has a higher potential to produce H_2_ than pectin during in vitro fermentation, due to its composition, and its total gas production was significantly higher than pectin [31]. This result is similar to that in this study; the gas production of FOS is significantly higher than that of PEC. In another study comparing the fermentation characteristics of FOS and BG, it was also found that FOS produced more gas than BG when fermented by gut microbiota [32].

Gas volume, pH, and SCFA production are all indices reflecting the degree of fermentation, and they are generally correlated with each other. The data of gas volume and pH (Figure 2B) indicated that CF and CA groups were the two most fully fermented groups. The more full fermentation of CA was also reflected in the SCFA production, while the SCFA production of CF is not consistent with its gas volume and pH, which may be due to the accumulation of SCFA intermediates (lactic acid) [33].

### 3.3. Total Carbohydrate Contents

Total carbohydrate content in fermentation broth at different fermentation times was determined through phenol–sulfuric acid method. In Figure 2C, the total carbohydrate content of each group with polysaccharide substrate generally decreased with increasing fermentation time. In general, utilization by gut microbiota was the highest for AX, followed by PEC and then BG; the utilization of FOS and PEC was mainly concentrated in the first 4 h of fermentation. Total carbohydrate contents in the N and the C groups were also about 1 mg/mL, which may be related to the carbohydrate from microbiota. FOS, PEC, BG, and AX are polysaccharides that can rapidly fermented in vitro static fermentation models [34]; thus, their degradation time is mainly concentrated in the first 12 h. The type and complexity of glycosidic linkages, monosaccharide molecular arrangement, molecular weight, particle size, solubility, and physical properties of DFs led to different fermentation rates and degrees of DFs in this study [34].

Polysaccharides were consumed in descending order: AX (85.2%) > PEC (68.3%) > BG (63.7%) > FOS (43.5%). Normally, the degradation of AX takes longer [35], but in this study, after 24 h, the degradation of AX was the most complete among the four polysaccharide groups. This may be due to the fact that the different preferences of microbiota in DFs degradation of different colonic locations [36] and different hosts’ dietary preferences, and leading to the highest degree of AX degradation.

### 3.4. Changes of C3G and C3G Metabolites during Fermentation

**Figure 3:**
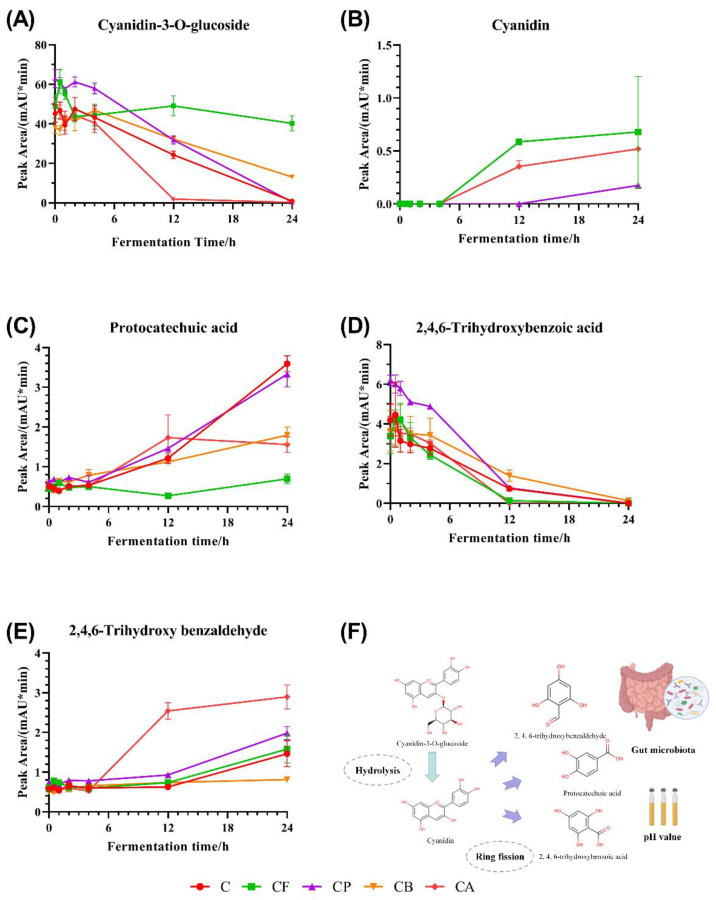
Changes in anthocyanin metabolites (**A**), Cyanidin-3-O-Glucoside (**B**), Cyanidin (**C**), PCA (**D**), 2,4,6-trihydroxybenzoic acid (TBA) (**E**), and (TB) during in vitro fermentation and the structural changes in metabolites (**F**).

We used HPLC to analyze the metabolites in fermentation broth of each group at each time point, compared to standard C3G metabolites. The results obtained are shown in Figure 3A. Figure 3A shows that the peak area for C3G decreased with the increasing of fermentation time in all the groups, but that the C3G was degraded and metabolized to varying degrees in the different groups. Notably, C3G was almost completely metabolized in C, CP, and CA groups after 24 h in vitro fermentation. However, there was still a high proportion of C3G in the fermentation broth of the CF group, and only 1/2 to 2/3 of the original C3G had been consumed in the CB group, indicating that the presence of FOS and BG may have a protective effect on C3G to some extent. In addition, C3G in group CA was the earliest to degrade among all the groups; this may be related to the high amount of *Lactobacillus* and *Bifidobacteria* in group CA [37].

Cyanidin is the hydrolyzed product of the C3G glycosidic bond [38] (Figure 3F). The changes in the peak area for cyanidin in six groups of samples at seven time points after integration are shown in Figure 3B. Cyanidin was detected in the fermentation broth in only three of the six groups. Generally speaking, the changes in C3G and cyanidin should be inversely proportional; that is, the more cyanidin breaks away from the C3G glycosidic bond, the more cyanidin will be retained in the fermentation broth. However, the C3G peak area for the CF group was still relatively high after 24 h, and this phenomenon was also observed for the cyanidin peak area in the corresponding integral graph. Therefore, we speculate that the co-fermentation of FOS and C3G may have protective effects on both C3G and cyanidin. Although the C3G in the CB group was not totally consumed by gut microbiota completely within 24 h, cyanidin was not detected in the fermentation broth, indicating that the protective effects of BG on cyanidin was not obvious. Most of the C3G in the CA group had been consumed before 12 h of culture, but cyanidin was still detected at 24 h, indicating that AX protected cyanidin from fermentation by microbiota.

Previous studies have investigated the metabolism of C3G in rats or in vitro fermentation model, and identified PCA, TBA, and TB as the major colonic metabolites [39,40] that were affected by gut microbiota and the pH value of the environment [38] (Figure 3F). Therefore, we integrated the peak area of PCA, TBA, and TB, which were the C3G metabolites in the fermentation broth. Figure 3C–E shows that PCA and TB did not significantly change during the 0–4 h phase, but did gradually increase after 4 h, which is in accordance with the degradation status of C3G after 4 h. The main metabolites in the CF group was cyanidin, and the main metabolites for CA were TB and cyanidin. These acidic metabolites may be absorbed by epithelial cells via monocarboxylic acid transporter [41].

Different polysaccharide substrates have different effects on the degradation of C3G by gut microbiota. FOS slowed down the degradation of C3G and prevented the further degradation of cyanidin. PEC modified the end product of C3G, so that the protocatechuic acid became the mainly metabolites. Similar to FOS, BG had the same inhibitory effect on C3G degradation, but the inhibitory effect is not as obvious as FOS, and the final main production was PCA. CA had the effect of promoting the degradation of C3G, which was almost degraded after 12 h of in vitro fermentation, and the main metabolite was TB. The different ways in which polysaccharides regulate C3G metabolism will help us to target those metabolites that are beneficial to health.

### 3.5. Alpha- and Beta-Diversity of Gut Microbiota

**Figure 4:**
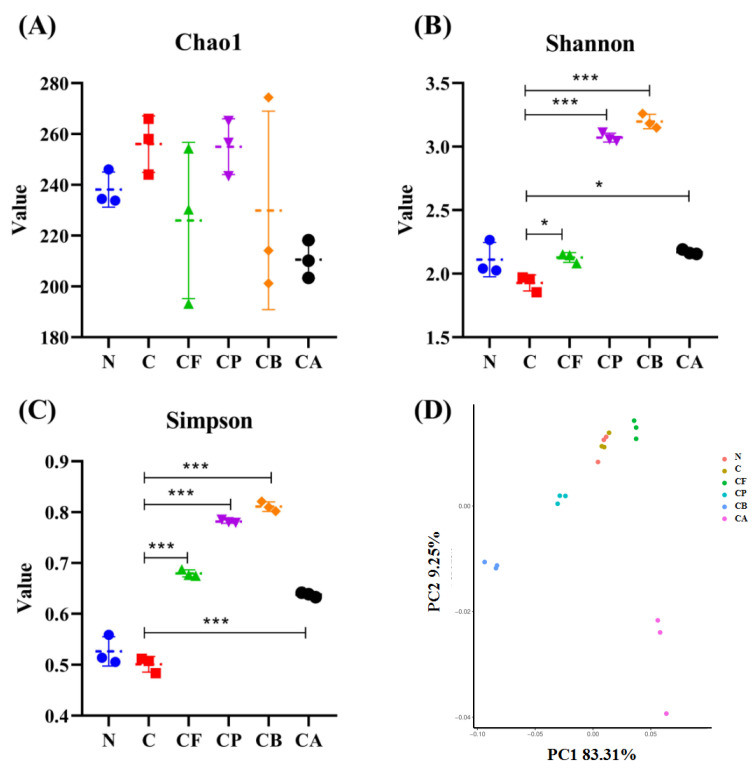
Analysis of different microbial α-diversity and β-diversity indices in the six experimental groups. The Chao1 (**A**) index was used as richness estimators. The Shannon (**B**) and Simpson (**C**) indices were used as diversity estimators. Principal Coordinates Analysis (PCoA) (**D**) was based on the weighted UniFrac distance matrix generated from all the samples in each group. Asterisks indicate significant difference between fermentation with C3G only and with C3G mixed with different polysaccharides according to an ordinary one-way ANOVA: * (*p* < 0.05), and *** (*p* < 0.001). Values are means ± SD for *n* = 3, as below.

In order to evaluate the impact of the different C3G-polysaccharide co-cultures on in vitro gut microbiota, the bacterial lineages of the in vitro fermentation broth were characterized. Alpha-diversity analysis was performed to reveal the diversity of species in each individual sample. The constructed dilution curves of diversity index show the difference in species richness. The Chao 1 (Figure 4A) index shows that there was no significant difference in species richness among the six experimental groups. Only the species richness in groups C and CP increased slightly; this was beneficial to the formation of a complete bacterial community structure. Both the Shannon (Figure 4B) and Simpson (Figure 4C) indices account for abundance and distribution of the species present. The addition of the four different polysaccharides to C3G significantly improved the diversity of gut microbiota after in vitro fermentation for 24 h, as indicated by the Shannon and Simpson indices, especially for PEC and BG.

Beta-diversity illustrates the differences between different populations. PCoA was used to further analyze the variation of the four polysaccharides mixed with C3G (Figure 4D). PCoA with Weighted Unifrac method showed that the first and second principal components explained 83.31% and 9.25% of the variation in microbial diversity, respectively. As can be seen in Figure 4D, the microorganisms in CF, CP, CB, and CA exhibited varying degrees of ability to utilize the substrate, indicating fine microbial differentiation due to the substrate chemistry. For PCoA, statistical analyses (ADONIS) showed that the four C3G-polysaccharide mixtures had a significant effect on the microbial community (*p* < 0.001), which further confirmed the important effect of dietary fiber type on microbial community structure [42]. In contrast to CF, CP, CB, and CA groups, the N and C groups clustered closely in the PCoA plot, indicating a significant similarity (low beta-diversity) between the two samples. These results indicate that C3G fermentation alone has little effect on changes in microbial community.

### 3.6. Microbial Community Distribution

**Figure 5:**
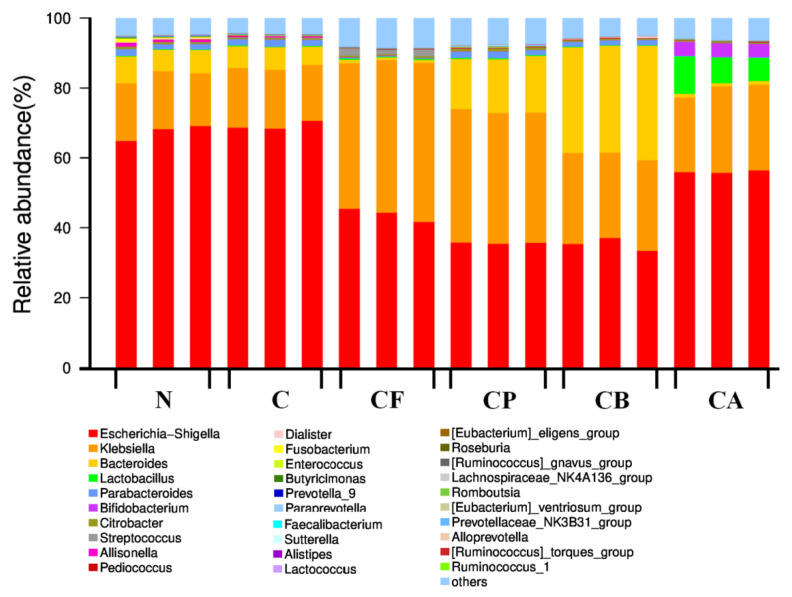
Relative abundance by genus level. Each column represents one sample. Different colors indicate different bacteria in genus.

Bacterial taxonomy at the genus level, at 24 h of incubation, with each substrate, is shown in Figure 5. We analyzed the sequencing results of each group, and Proteobacteria and Bacteroidetes were the dominant bacteria detected in all samples. The dominant microbiota at the genus level were *Escherichia–Shigella*, *Klebsiella*, *Bacteroides*, and *Lactobacillus*. In order to further determine the difference in microbial community among the six groups at the genus level, statistical analyses were conducted on the dominant species at the genus level.

**Figure 6:**
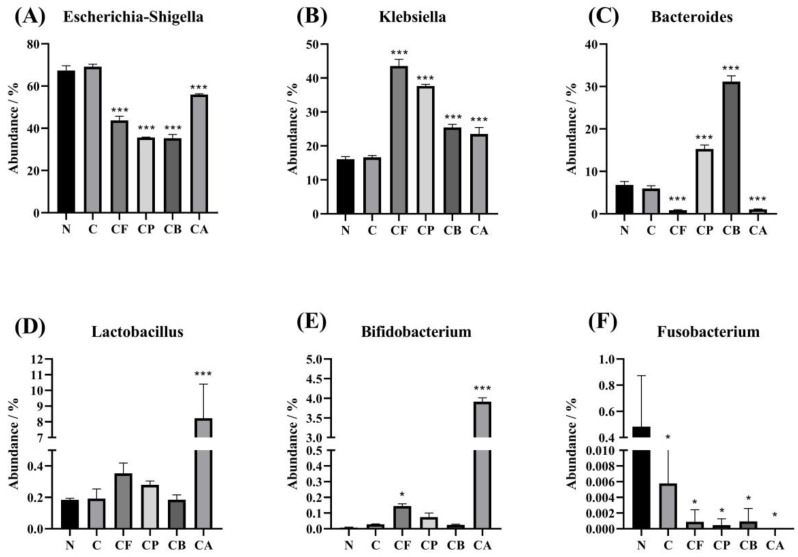
Significant differences among the five substrates and the control at the genus level. Asterisks indicate significant difference between N group and other five groups, respectively: * (*p* < 0.05), and *** (*p* < 0.001). Values are means ± SD for *n* = 3.

We performed an analysis of ANOVA and counted six different abundant microbiomes at the genus level (Figure 6). *Escherichia–Shigella* could impact the health of the host, leading to an unstable gut microbiota associated with low-grade inflammation or even cause chronic colitis. Thus, lower levels of fecal *Escherichia–Shigella* are likely to be beneficial. In this study, the *Escherichia–Shigella* group predominated during fermentation, possibly due to the presence of residual amounts of oxygen during inoculum activation. However, at the same time, we noticed that the four C3G-polysaccharide complexes all reduced the abundance of *Escherichia–Shigella* (Figure 6A) in the fermentation samples to varying degrees, which is beneficial to the health of the GIT. This observation could be attributed to the fact that the fermentation of polysaccharides resulted in an acidic environment that was not suitable for the growth of *Escherichia–Shigella*. Both *Bacteroides* (Figure 6C) and *Lactobacillus* (Figure 6D) can induce an acidic environment [43], which could explain why the abundance of *Escherichia–Shigella* in CB and CP groups was lower than that in the other two groups (CF and CA). *Lactobacillus* species have significant effects on intestinal permeability, inflammation, body weight, and obesity [44,45]. A high presence of *Bifidobacterium* is inversely associated with increased fat mass, glucose tolerance, adipose tissue inflammation, and lipopolysaccharide (LPS) levels [46]. In our study, the abundance of *Lactobacillus* (Figure 6D) and *Bifidobacterium* (Figure 6E), two types of beneficial bacteria commonly found in gut microbiota, was improved; this was consistent with previous a study [17]. The four polysaccharides and C3G have some degree of probiotic-growth-promoting effect. In addition, the compound substance C3G–AX significantly increased the abundance of *Lactobacillus* and *Bifidobacterium*, and was apparently higher than that of other groups. Previous studies have demonstrated a more bifidogenic effect for AX than BG [35]. An increase in *Bifidobacterium* and *Lactobacillus* induced by anthocyanins had also been previously reported [47,48]. C3G could be degraded by β-glucosidases secreted by the microbiota to provide energy and more suitable pH for the growth of the probiotics [17]. However, since we did not set up a pure polysaccharide group, it is not clear whether this effect of promoting probiotic growth is synergistic. *Fusobacterium* positively correlated with fasting blood glucose, glycosylated serum protein, LPS, insulin resistance index (HOMA-IR), and three inflammatory factors (TNF-alpha, IL-1 beta, and IL-6) in rats [49]. We compared the abundance of *Fusobacterium* (Figure 6F) in our six groups after 24 h of in vitro fermentation. Our results showed that C3G alone and the different C3G–polysaccharide complexes significantly decreased the abundance of *Fusobacterium*, and that the polysaccharides enhanced the effect of C3G to a similar extent.

### 3.7. SCFAs Production

The main SCFAs in the gut, including acetic acid, propionic acid, and butyric acid, are generally considered to be beneficial for human health. The SCFA plays a crucial role in maintaining the barrier function of the gut, regulating epithelial proliferation, preventing colorectal cancer, and modulating immune responses [50,51]. Therefore, accurately determining the level of SCFAs is important for evaluating the fermentation behavior of C3G mixed with dietary fibers in the gut. However, in vivo produced SCFAs are difficult to measure, since more than 95% of SCFAs are rapidly absorbed and metabolized by the GIT [52]. For this reason, the concentrations of SCFAs in fecal samples do not truly represent those in the proximal colon. Compared with in vivo methods, in vitro batch fermentation is a more promising approach that can be adapted to determine the actual fermentation behavior of polysaccharides more accurately [34]. Thus, we employed batch fermentation and used gas chromatography to determine SCFAs. To study the effects of in vitro fermentation on SCFA production in C3G mixed with four different polysaccharide treatments, we measured the concentrations of acetic acid, propionic acid, butyric acid, valeric acid, isobutyric acid, and isovaleric acid.

**Figure 7:**
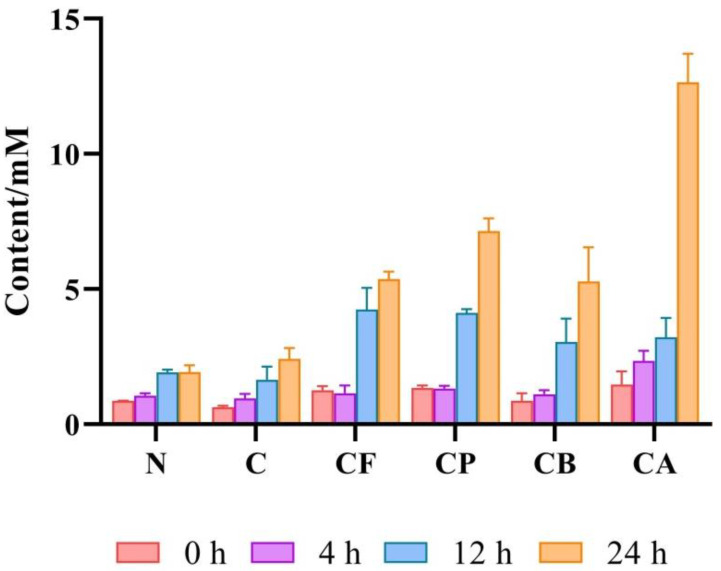
Total C3G SCFAs mixed with different polysaccharides during in vitro fermentation.

Total SCFA concentrations in the fermentation broth (Figure 7) increased gradually with the extension of fermentation time. Compared with the N group, SCFA concentrations in the fermentation broth for C3G only and C3G mixed with different polysaccharides were higher. This indicates that both C3G alone and C3G mixed with different polysaccharides can promote SCFA production by gut microbiota. SCFAs are derived from microbial fermentation of dietary fibers [53], so the total SCFA production of the four groups with polysaccharide substrates was significantly higher than the other two groups (N and C3G alone). Total SCFA concentrations in the fermentation broth for group C were also higher than for group N; however, the effect was not significant. This could be explained by the breakdown of the glucoside bonds in C3G by bacterial enzymes, resulting in the situation that microbiota used the glucose to produce SCFAs [54].

**Figure 8:**
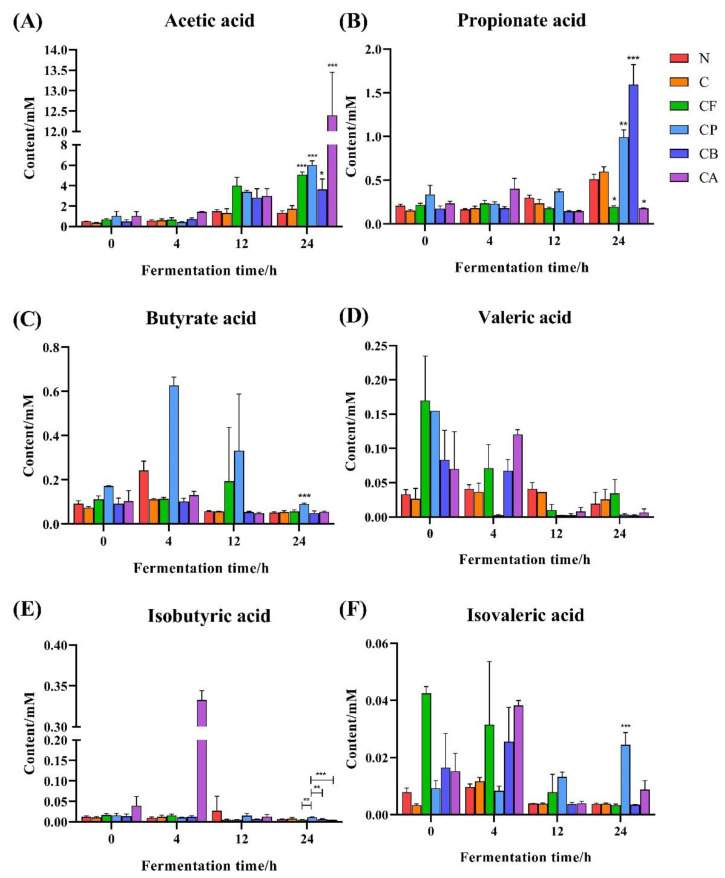
Effects of C3G mixed with different polysaccharides on SCFAs during in vitro fermentation: (**A**) acetic acid, (**B**) propionic acid, (**C**) butyric acid, (**D**) valeric acid, (**E**) isobutyric acid, and (**F**) isovaleric acid. Asterisks indicate significant difference between N group and other five groups at 24 h of fermentation, respectively, except for isobutyric acid: * (*p* < 0.05), ** (*p* < 0.01) and *** (*p* < 0.001). Values are means ± SD for *n* = 3

In this study, the concentrations of acetic acid (Figure 8A) in the fermentation broth were the highest, and they gradually increased with the extension of the fermentation time. At 24 h of fermentation, the acetic acid concentrations in the fermentation broths with a polysaccharide substrate were significantly higher than for the N and C groups. In particular, acetic acid production in the CA group was significantly higher than that of all the other groups. This may be because the combination of C3G and AX increases the abundance of *Bifidobacterium*, and acetic acid is the final product of *Bifidobacterium*. Acetic acid can enter the blood circulation system and be oxidized by the brain, heart, and peripheral tissues [55]. Acetic acid may be closely related to the regulation of body weight, because high concentration of acetic acid can promote the expression of AMP kinase and inhibit the synthesis of fat [56]. Therefore, the combination of C3G and AX may be more effective than other combinations in controlling weight gain.

Propionic acid (Figure 8B) produced the second highest SCFA concentrations in our study. Surprisingly, combining C3G with FOS and AX did not increase propionic acid production. However, mixing C3G with PEC and BG did significantly increase propionic acid production in the fermentation broth, especially for the CB group. Previous studies have similar results to ours [30,43]. A significant increase in the Bacteroides (Figure 5E) from β-glucan fermentation may explain the increase in propionate production [57]. Propionic acid is absorbed by the liver, participates in gluconeogenesis, and inhibits cholesterol synthesis [58], so we hypothesize that the combination of C3G and BG has the potential to lower cholesterol.

Butyric acid (Figure 8C) is an important source of energy for intestinal epithelial cells; can regulate the growth and apoptosis of epithelial cells and immune cells; can inhibit colitis and colon cancer; and can play a crucial role in maintaining the integrity of the intestinal mucosal barrier and in stabilizing the intestinal microenvironment [59]. Mixing C3G and PEC significantly increased butyric acid production during in vitro fermentation, with levels peaking at 4 h. This may be due to the fact that pectin promotes the growth of *Faecalibacterium* and *Ruminococcaceae* during fermentation [60], thus increasing butyric acid production, which is subsequently consumed as an energy source by microbiota. The results of Harris et al. on butyric acid production of different DFs in the mid fermentation were similar to ours [61]. However, in the mid-late fermentation, β-glucan and FOS did not produce significantly higher butyric acid, as shown in a previous study [62], which may due to the changes the metabolic preference of microorganisms affecting by C3G. In addition, the amounts of isobutyric, isovaleric, and valeric acid (Figure 8D–F) were very low. Indeed, few bacteria are known to produce these three acids [63].

## 4. Conclusions

In conclusion, the used dietary fibers modulate the fermentation patterns of cyanidin-3-*O*-glucoside in a fiber-type-dependent manner. Depending on its structure, each compound provided a specific carbon source for the fermentation of different bacterial populations, thereby influencing the beneficial taxa and producing different amounts of SCFAs and gases in vitro. The present work illustrated the interaction among anthocyanins, polysaccharides, and gut microbiota, which could provide a reference for elucidating the health effects of the supplementation of C3G-polysaccharide diets. For future research, the individual dietary fiber groups will be included to study the potential synergistic effects of C3G and dietary fiber on fermentation patterns.

## Data Availability

Data available in a publicly accessible repository.
